# Supplementary material for: Identification of Freezing-Responsive microRNAs and Their Targets in Chinese Jujube by Small RNA and Degradome Sequencing
Source: Int J Mol Sci. 2026 Sep 4;27(17):7912. doi: 10.3390/ijms27177912 (PMC13566890; doi:10.3390/ijms27177912)
Supplement: Supplementary file 1 [file ijms-27-07912-s001.zip › Supplementary Material S2/novel_miR_221.pdf]

Provisional ID	: novel_miR_221	
Score total	:	0.4
Score for star read(s)	:	-1.3
Score for read counts	:	0
Score for mfe	:	0.1
Score for randfold	:	1.6
Score for cons. seed	:	
Total read count	:	57
Mature read count	:	57
Loop read count	:	0
Star read count	:	0

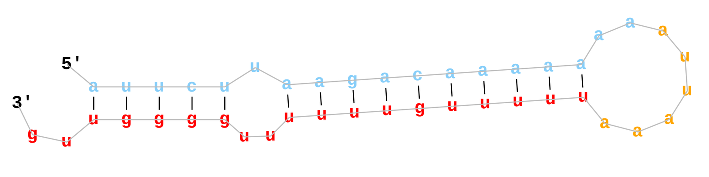

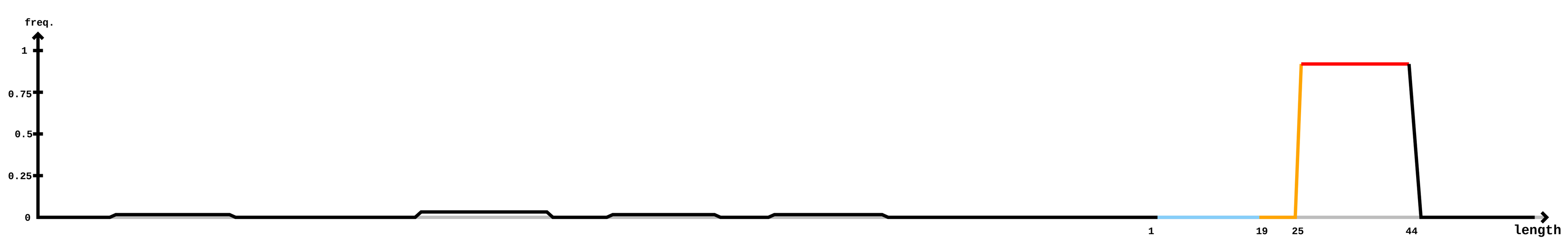

Star Mature

```
5'- gacuaaacgggaaaaaaaaaaaaaagagaagcgaaaugcaggguuuaggguuuggauuccaugaauauguggaagcuuuagaagaauggagaaagauugaggauuuucaaagagagcuuccucuauguuuggagcuuguggcacaaacuuuguaaaaaaaaagacccuucuugaucuucagcaugauucuuaagacaaaaaaaauuaaauuuuuguuuuuugggguuggauaucugauugguugugguuu   -3'    exp
    ((((((.((((..................(((((...(((((...(((((((...((((((.....)))))).(((((......))))).......(((((..(((.....)))..)))))...(((((..(((...))).))))).............))))))))))))...))).)).((((((.((((((((((.......))))))))))..)))))))....)))).)))))).......      reads    mm    sample
    ..........aaaaaGaaaaaaaaagagaag.........................................................................................................................................................................................      1        1     LT-3
    ..........................................................gaGuauguggaagcuuuagaag..........................................................................................................................................      2        1     JCK-1
    ..........................................................................................gauuCaggauuuucaaag................................................................................................................      1        1     JCK-1
    ......................................................................................................................ucuGuguuuggagcuugug.......................................................................................      1        1     JCK-3
    .....................................................................................................................................................................................uuuuuguuuuuugggguug.....................      26       0     LCK-2
    .....................................................................................................................................................................................uuuuuguuuuuugggguug.....................      31       0     LCK-1
```
